# Radiological‐histopathological discordance in patients transplanted for HCC and its impact on post‐transplant outcomes

**DOI:** 10.1002/cam4.6161

**Published:** 2023-06-16

**Authors:** Islam B. Mohamed, Mohamed Saleh Ismail, Ahmed El Sabagh, Ahmed M. Afifi Abdelwahab, Efstathia Polychronopoulou, Yong‐Fang Kuo, Manal Hassan, John A. Goss, Fasiha Kanwal, Prasun K. Jalal

**Affiliations:** ^1^ Section of Gastroenterology and Hepatology Baylor College of Medicine Houston Texas USA; ^2^ Department of Epidemiology The University of Texas MD Anderson Cancer Center Houston Texas USA; ^3^ Department of Biostatistics The University of Texas Medical Branch Galveston Texas USA; ^4^ Division of Abdominal Transplantation Baylor College of Medicine Michael E. DeBakey Department of Surgery Houston Texas USA

**Keywords:** explant pathology, HCC recurrence, hepatocellular carcinoma, liver transplantation, OPTN/UNOS, survival after LT

## Abstract

**Background and Aims:**

Contrast‐enhanced cross‐sectional imaging is the cornerstone in the diagnosis, staging, and management of HCC, including eligibility for liver transplantation (LT). Radiological‐histopathological discordance may lead to improper staging and may impact patient outcomes. We aimed to assess the radiological‐histopathological discordance at the time of LT in HCC patients and its impact on the post‐LT outcomes.

**Methods:**

We analyzed further the effect of 6‐month waiting policy on the discordance. Using United Network for Organ Sharing—Organ Procurement and Transplantation Network (UNOS‐OPTN) database, we examined the discordance between pre‐LT imaging and explant histopathology for all adult HCC patients who received liver transplants from deceased donors between April 2012 and December 2017. Kaplan–Meier methods and Cox regression analyses were used to evaluate the impact of discordance on 3‐year HCC recurrence and mortality.

**Results:**

Of 6842 patients included in the study, 66.7% were within Milan criteria on both imaging and explant histopathology, and 33.3% were within the Milan based on imaging but extended beyond Milan on explant histopathology. Male gender, increasing numbers of tumors, bilobar distribution, larger tumor size, and increasing AFP are associated with increased discordance. Post‐LT HCC recurrence and death were significantly higher in patients who were discordant, with histopathology beyond Milan (adj HR 1.86, 95% CI 1.32–2.63 for mortality and 1.32, 95% CI 1.03–1.70 for recurrence). Graft allocation policy with 6‐month waiting time led to increased discordance (OR 1.19, CI 1.01–1.41), although it did not impact post‐LT outcome.

**Conclusion:**

Current practice for staging of HCC based on radiological imaging features alone results in underestimation of HCC burden in one out of three patients with HCC. This discordance is associated with a higher risk of post‐LT HCC recurrence and mortality. These patients will need enhanced surveillance to optimize patient selection and aggressive LRT to reduce post‐LT recurrence and increase survival.

## Graphical abstract

Contrast‐enhanced Imaging is the cornerstone in the diagnosis, staging, and decision for LT in patients with HCC. In this study of nearly 7000 patients, there was a discrepancy between the imaging and explanted liver in tumor size, number, and presence of vascular invasion in one of three patients. Male gender, increasing numbers of tumors, bilobar distribution, larger tumor size, and increasing AFP were associated with increased discrepancy. There was increased mortality and recurrence after liver transplantation in this group. Six‐month waiting policy after listing for transplantation led to increased discordance.

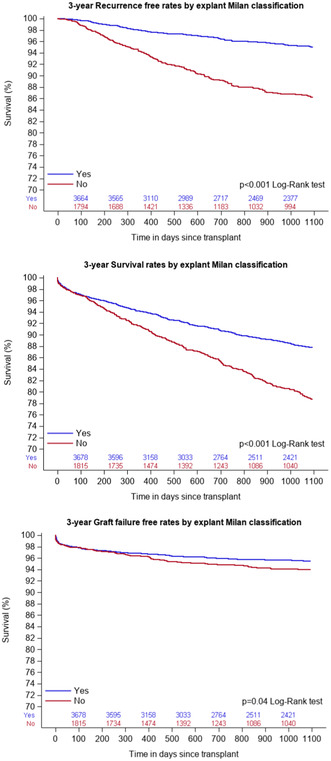

## 1 INTRODUCTION

Hepatocellular carcinoma (HCC) is the fourth most common cause of cancer‐related deaths worldwide.^1^ According to the latest report from the Surveillance, Epidemiology, and End Results (SEER) data, the incidence of HCC has significantly increased over the last three decades. Moreover, the incidence of HCC is estimated to rise by 2.8% per year by 2030, and HCC has become a leading indication for liver transplantation (LT) in the USA.^2^ However, starting 2013 the incidence started to decrease by 3.2% per year.^3^

American Association for the Study of Liver Diseases (AASLD) recommends contrast‐enhanced imaging to establish the diagnosis of HCC and limits biopsy only for patients with indeterminate lesions.^4^ The Milan criteria based on radiology alone are the benchmarks used for listing of HCC patients for LT and maintenance on wait‐list.^5^ Few available studies showed a discordance between radiological and histopathological findings in patients undergoing LT; as many as 40% had occult lesions on explant histopathology missed in pre‐LT radiological assessment.^6^ However, there is a lack of information on the effect of such discordance on patient outcome following LT.

The 6‐month waiting policy (wait and cap) after listing was implemented in October 2015 to balance the access for LT between HCC and non‐HCC patients.^7^ It also allowed time to observe biological behavior of HCC and avoiding LT in patients with aggressive tumor.^8^ However, the effect of implementation of the policy on the radiological‐histopathological discordance has not been explored.

In this study, using a large national database, we analyzed data of transplanted HCC patients to assess the radiological‐histopathological discordance at time of LT and its impact on the post‐LT outcomes. We analyzed further the effect of 6‐month waiting policy on the discordance and post‐LT outcomes. Secondary aim was to explore the difference between contrast‐enhanced liver protocol imaging (CT and MRI) in predicting radiological‐histopathological discordance.

## 2 PATIENTS AND METHODS

### 2.1 Study design

**FIGURE 1:**
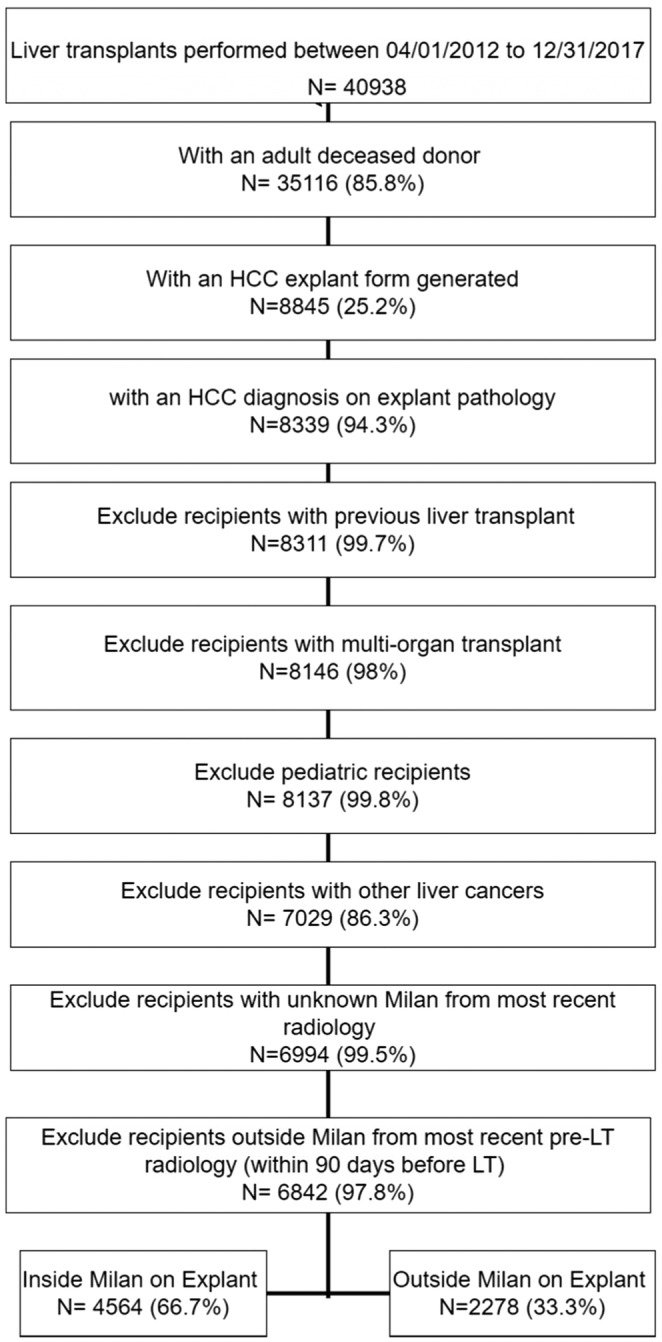
Flowchart of study cohort selection.

We retrospectively analyzed data for adult patients (> = 18 years) with deceased donor liver‐alone transplants in the UNOS/OPTN STAR (Standard Transplant Analysis and Research) file between April 1, 2012 and December 31, 2017 and an explant form confirming the diagnosis of HCC based on histopathology. UNOS started to incorporate data from explant pathology starting in April 2012. Figure 1 explains the inclusion criteria for analyses. We limited our study to patients who had documented contrast‐enhanced cross‐sectional imaging within 90 days prior to LT that showed tumor within the Milan criteria. We chose imaging within 90 days of transplant because HCC patients on transplant wait‐list are followed with imaging every 3 months.

The 6‐month waiting policy for HCC patients was implemented in October 2015 to optimize organ allocation, and we tested the effect of the policy on radiology‐histopathology discordance and post‐LT outcomes.

### 2.2 Study variables

We analyzed recipient characteristics including age, gender, race/ethnicity, and body mass index (BMI). Patients' laboratory and clinical data included MELD score, waiting time on a transplant list, alpha‐fetoprotein (AFP), Trans‐jugular intrahepatic portosystemic shunt (TIPSS), spontaneous bacterial peritonitis (SBP), previous abdominal surgeries, number and type of down‐staging treatments received, and other comorbidities. We also collected data for donor characteristics including age, gender, race/ethnicity, BMI, cold ischemia time, comorbidities, HCV (Hepatitis C virus) Antibody (HCV Ab), and hepatitis B core Ab (HBcAb) status.

To explore discordance, we collected data for explant pathology parameters including tumor differentiation, number of tumors, vascular invasion, extrahepatic spread, satellite lesions, and lymph nodes involvement. A contrast‐enhanced imaging study with MRI (magnetic resonance imaging) or CT scan within 90 days pre‐LT was analyzed to assess the number of tumors, tumor size, and extrahepatic spread. We defined radiology‐histopathological discordance as the disagreement between pre‐LT radiology and explant histopathology regarding number of tumors, size of tumors, and presence of vascular invasion.

### 2.3 Study Outcomes

We tested the effect of discordance on post‐LT outcomes that includes 3‐year mortality, tumor recurrence (defined of radiological recurrence of HCC intra or extrahepatic), and graft failure (defined as graft nonfunction with deterioration of liver synthetic function). We analyzed the effect of 6‐month waiting policy on the discordance and post‐LT outcomes. We also explored the difference between contrast‐enhanced imaging by CT and MRI to detect the discordance between radiology and explant histopathology.

This study received Institutional Review Board approval from Baylor College of Medicine, Houston, Texas. Data collected through UNOS/OPTN STAR file comply with relevant data protection and privacy regulations.

### 2.4 Statistical analysis

Clinical and tumor characteristics were described in medians and interquartile ranges (IQRs) or frequency and percentages. Patient and tumor characteristics were compared between the explant pathology and the most recent pretransplant radiology with the use of chi‐square, *t*‐test, or nonparametric tests as appropriate to assess statistical differences. We used Kaplan–Meier method to determine the HCC recurrence, graft failure, and mortality at 3 years.

Multivariable Cox proportional hazards models were used to assess the effect of being outside Milan at explant on the post‐LT outcomes while adjusting for patient clinical, and tumor characteristics. All patients were censored at the loss of follow‐up, the end of the follow‐up period (3 years), or at the end of the study period (12/31/2018), whichever occurred first. In the analysis of graft failure and HCC recurrence, patients were also censored at death if it occurred first. A multivariable model was constructed with all significant variables from the univariate analyses. Baseline demographics and clinical characteristics and HCC characteristics were added to the final model regardless of their significance in the univariate analyses.

We created a logistic regression analysis to study the factors associated with discordance. A multivariable logistic regression model was constructed to determine the effect of 6‐month waiting policy on the risk of being outside Milan at explant. All analyses were conducted using SAS 9.4 (Cary, NC, USA).

## 3 RESULTS

### 3.1 Cohort characteristics (donor/recipient)

A total of 6842 adult patients underwent LT for HCC with imaging inside Milan within 90 days pre‐LT. Table 1 Patients were classified into groups according to concordance between pre‐LT (within 90 days) images and explant histopathology. Group 1 included 4564 (66.7%) patients who were categorized as within Milan criteria by both radiology and explant histopathology. Group 2 included 2278 (33.3%) patients who were within Milan criteria by radiology but outside Milan by explant histopathology.

**TABLE 1:** Discordance between radiology (within 3 months pretransplant) and explant histopathology in the study population.

Radiology	Explant			*p* value
	Milan: Yes (%)	Milan: No (%)	Total	
Milan: Yes	4564 (66.7) Group 1	2278 (33.3) Group 2	6842	<0.0001
Milan: No	62 (40.8) Group 3	90 (59.2) Group 4	152	
Total	4626	2368	6994	

The baseline characteristics of 6842 transplant recipients with pre‐LT imaging within Milan are summarized in Table 2. For recipients' characteristics, 80% of the recipients were males, and 67% were of white ethnicity. At transplant, 87% of the patients had high or moderate functional status, 65% of the patients had AFP ≤20 ng/mL, and 16.5% of the patients had ≥ one locoregional treatment while on a transplant waiting list. Hepatitis C (HCV), nonalcoholic steatohepatitis (NASH), and Alcoholic liver disease (ALD) were leading HCC etiologies representing 62%, 11%, and 9%, respectively.

**TABLE 2:** Characteristics of transplanted patients with HCC within Milan by pre‐LT radiology (Group 1 and 2).

Characteristics	Category	Total	Group 1 *N* = 4564 (%)	Group 2 *N* = 2278 (%)	*p* value
Donor characteristics					
Donor gender	F	2758	1853 (40.6)	905 (39.7)	0.49
	M	4084	2711 (59.4)	1373 (60.3)	
Deceased donor cause	Anoxia	2421	1585 (34.7)	836 (36.7)	0.38
	CVA	2372	1601 (35.1)	771 (33.8)	
	Trauma	1890	1275 (27.9)	615 (27)	
	Other	159	103 (2.3)	56 (2.5)	
Liver type	Partial/split	70	50 (1.1)	20 (0.9)	0.40
	Whole	6772	4514 (98.9)	2258 (99.1)	
Share type	Local	5324	3568 (78.2)	1756 (77.1)	0.59
	Regional	1305	857 (18.8)	448 (19.7)	
	National	213	139 (3)	74 (3.2)	
DCD (donor after cardiac death) donor	No	6327	4241 (92.9)	2086 (91.6)	0.04
	Yes	515	323 (7.1)	192 (8.4)	
ECD (expanded criteria donors) donor	No	4902	3261 (71.5)	1641 (72)	0.61
	Yes	1940	1303 (28.5)	637 (28)	
Recipient characteristics					
Recipient gender	F	1394	1018 (22.3)	376 (16.5)	**<0.0001**
	M	5448	3546 (77.7)	1902 (83.5)	
Ethnicity	White	4583	3080 (67.5)	1503 (66)	0.60
	Black	686	445 (9.8)	241 (10.6)	
	Hispanic	1013	670 (14.7)	343 (15.1)	
	Other	560	369 (8.1)	191 (8.4)	
Education	Grade school or lower	419	267 (5.9)	152 (6.7)	0.44
	High school	3019	2026 (44.4)	993 (43.6)	
	College	1686	1127 (24.7)	559 (24.5)	
	More than college	1478	975 (21.4)	503 (22.1)	
	Unknown	240	169 (3.7)	71 (3.1)	
Region	Central	1384	919 (20.1)	465 (20.4)	**<0.0001**
	Northeast	1795	1192 (26.1)	603 (26.5)	
	South	2178	1538 (33.7)	640 (28.1)	
	West	1485	915 (20)	570 (25)	
Functional status at transplant	Unknown	52	30 (0.7)	22 (1)	**<0.0001**
	Low	871	570 (12.5)	301 (13.2)	
	Moderate	3664	2514 (55.1)	1150 (50.5)	
	High	2255	1450 (31.8)	805 (35.3)	
Previous malignancy	No	1541	1038 (22.7)	503 (22.1)	0.82
	Yes	3536	2354 (51.6)	1182 (51.9)	
	Unknown	1765	1172 (25.7)	593 (26)	
Diabetes	No	4575	3061 (67.1)	1514 (66.5)	0.64
	Yes	2185	1452 (31.8)	733 (32.2)	
	Unknown	82	51 (1.1)	31 (1.4)	
AFP	<=20 ng/mL	4462	3142 (68.8)	1320 (57.9)	**<0.0001**
	21–99 ng/mL	992	608 (13.3)	384 (16.9)	
	100–499 ng/mL	396	222 (4.9)	174 (7.6)	
	> = 500 ng/mL	88	48 (1.1)	40 (1.8)	
	Unknown	904	544 (11.9)	360 (15.8)	
Previous abdominal surgery	No	3652	2399 (52.6)	1253 (55)	0.12
	Yes	3148	2134 (46.8)	1014 (44.5)	
	Unknown	42	31 (0.7)	11 (0.5)	
Port. vein thrombosis	No	5916	3942 (86.4)	1974 (86.7)	0.93
	Yes	906	609 (13.3)	297 (13)	
	Unknown	20	13 (0.3)	7 (0.3)	
TIPS	No	6366	4259 (93.3)	2107 (92.5)	0.29
	Yes	417	264 (5.8)	153 (6.7)	
	Unknown	59	41 (0.9)	18 (0.8)	
Acute rejection episode	Yes, treated	170	103 (2.3)	67 (2.9)	0.21
	Yes, not treated	56	39 (0.9)	17 (0.7)	
	No	6616	4422 (96.9)	2194 (96.3)	
Number of treatments since listing	0	4571	3178 (69.6)	1393 (61.2)	**<0.0001**
	1	1142	767 (16.8)	375 (16.5)	
	>1	1129	619 (13.6)	510 (22.4)	
Number of treatments since most recent radiology	0	6268	4207 (92.2)	2061 (90.5)	**0.02**
	1 or 2	574	357 (7.8)	217 (9.5)	
Thermal ablation since most recent radiology	No	6763	4523 (99.1)	2240 (98.3)	0.06
	Yes	79	41 (0.9)	38 (1.7)	
Chemoemb. since most recent radiology olisation	No	6400	4276 (93.7)	2124 (93.2)	0.48
	Yes	442	288 (6.3)	154 (6.8)	
Approved HCC exception ever	No	239	132 (2.9)	107 (4.7)	**<0.0001**
	Yes	6603	4432 (97.1)	2171 (95.3)	
Hepatitis B core antibodies	Negative	4349	2936 (64.3)	1413 (62)	**0.01**
	Positive	2274	1469 (32.2)	805 (35.3)	
	Unknown/not disclosed	219	159 (3.5)	60 (2.6)	
Hep B surface antigen	Negative	6270	4174 (91.5)	2096 (92)	**0.01**
	Positive	402	259 (5.7)	143 (6.3)	
	Unknown/not disclosed	170	131 (2.9)	39 (1.7)	
HIV serostatus	Negative	6568	4391 (96.2)	2177 (95.6)	0.41
	Positive	38	25 (0.5)	13 (0.6)	
	Unknown/not disclosed	236	148 (3.2)	88 (3.9)	
HCV serostatus	Negative	2249	1477 (32.4)	772 (33.9)	**0.001**
	Positive	4468	2984 (65.4)	1484 (65.1)	
	Unknown/not disclosed	125	103 (2.3)	22 (1)	
HCC etiology	ALD	668	456 (10)	212 (9.3)	0.50
	Crypto	165	109 (2.4)	56 (2.5)	
	HBV	415	266 (5.8)	149 (6.5)	
	HCV	4285	2872 (62.9)	1413 (62)	
	HCV_ALD	584	395 (8.7)	189 (8.3)	
	NASH	725	466 (10.2)	259 (11.4)	
Time from imaging to transplant in days	Median(IQR)	6842	35 (15–58)	35 (16–61)	0.77
Tumor characteristics					
Number of tumors at most recent radiology	1	5134	3565 (78.1)	1569 (68.9)	**<0.0001**
	2	608	362 (7.9)	246 (10.8)	
	3	199	95 (2.1)	104 (4.6)	
	0	901	542 (11.9)	359 (15.8)	
Number of tumors at explant	1	3449	3117 (68.3)	332 (14.6)	**<0.0001**
	2	1591	961 (21.1)	630 (27.7)	
	3	808	486 (10.6)	322 (14.1)	
	4	435	0 (0)	435 (19.1)	
	5	220	0 (0)	220 (9.7)	
	>5	299	0 (0)	299 (13.1)	
	Infiltrative	40	0 (0)	40 (1.8)	
Tumor location	1‐Left	1083	914 (20)	169 (7.4)	**<0.0001**
	2‐Right	4124	3077 (67.4)	1047 (46)	
	3‐Both	1595	573 (12.6)	1022 (44.9)	
	4‐Infiltrative	40	0 (0)	40 (1.8)	
Worst tumor differentiation	Complete tumor necrosis	1431	1119 (24.5)	312 (13.7)	**<0.0001**
	Poor	516	272 (6)	244 (10.7)	
	Moderate	3292	2061 (45.2)	1231 (54)	
	Well	1603	1112 (24.4)	491 (21.6)	
Vascular invasion	Microvascular	954	497 (10.9)	457 (20.1)	**<0.0001**
	Macrovascular	126	0 (0)	126 (5.5)	
	None	5762	4067 (89.1)	1695 (74.4)	
Lymph node involvement	No	6711	4564 (100)	2147 (94.2)	**<0.0001**
	Yes	131	0 (0)	131 (5.8)	
Extrahepatic spread	No	6805	4564 (100)	2241 (98.4)	**<0.0001**
	Yes	37	0 (0)	37 (1.6)	
Satellite lesions	No	6423	4384 (96.1)	2039 (89.5)	**<0.0001**
	Yes	419	180 (3.9)	239 (10.5)	

Significant *p* values are indicated in bold.

### 3.2 Tumor characteristics

Fifty percent of patients had more than one tumor on explant pathology. More tumors were found on right lobe of the liver, 48% and 23% of the tumors were moderately or well differentiated, respectively, and vascular invasion was found in 16% of the explants. Table 3 outlines the discordance in tumor characteristics between pre‐LT radiology and explant histopathology in further details. Histopathology showed a higher number, increased total diameter, and largest tumor size compared with radiology (*p* < 0.0001). Table S1 Among patients with discordant data, Microvascular invasion in explant histopathology was found in significantly higher no. of cases in group 2 (25.6%, *n* = 583) compared with group 1 (10.9%, *n* = 497), *p* < 0.0001. Macrovascular invasion was noted in histopathology in 5.5% (*n* = 126) patients described within Milan by pre‐LT radiology. No statistical difference was found between the time elapsed from last imaging to receipt of liver transplantation with a median (IQR) of 35 days (15–58) for group 1 and 35 days (16–61) in group 2, *p* = 0.77. Table 2.

**TABLE 3:** Agreement between the explant histopathology and pre‐LT imaging in the study population of 6994 patients.

	Within Milan		*p* value	Outside Milan		*p* value
	According to radiology	According to explant		According to radiology	According to explant	
Number of tumors, mean (SD)	1.00 (0.53)	1.42 (0.68)	<0.0001	1.04 (0.67)	3.21 (1.62)	<0.0001
Total size of tumors, mean (SD)	2.59 (1.10)	2.83 (1.29)	<0.0001	2.90 (1.30)	7.16 (3.02)	<0.0001
Largest tumor size, mean (SD)	2.59 (1.11)	2.32 (1.00)	<0.0001	2.94 (1.34)	4.10 (2.03)	<0.0001
Vascular invasion, *N* (%)	N/A	497 (10.89)	–	N/A	583 (25.6)	–

### 3.3 Predictors of discordance: risk factors for outside Milan on explant

Due to a change in allocation policy with mandatory 6‐month wait time before getting exception point in October 2015, we created a model testing the impact of this policy on the likelihood of being outside Milan at explant. Patients listed before October 2015 but transplanted afterwards were not included in this model, resulting in a total of 5783 patients (4328 before and 1455 after October 2015). After the implementation of the new policy, more patients were transplanted with an explant outside Milan 36.4% versus 31.8% before the policy, *p* < 0.0015. Table S2 Furthermore, there was a decrease in transplantation of HCC patients across all regions after the policy. Regardless of the volume of transplanted HCC patients, Northeast showed statistically significant difference in discordance rate before and after implementation of the policy *p* = 0.001. Table S3.

We examined factors associated with discordant results to patients who were listed and transplanted according to the same allocation policy in this model. On the multivariable analysis male gender, higher number of tumors, larger tumor size, and multifocality of tumors were associated with increased discordance. Table 4 Increased AFP compared with a lower level (≤ 20 ng/mL) was a predictor of being outside Milan at explant and the risk increased consistently with a higher AFP level (AFP 21–99 ng/mL, HR 1.48, 95% CI 1.26–1.74; AFP 100–499 ng/mL, HR 1.63, 95% CI 1.28–2.07; and AFP ≥500 ng/mL, HR 2.4, 95% CI 1.49–3.864). Patients who received more than one locoregional treatment (LRT) of HCC pre‐LT were more likely to be outside Milan compared with patients receiving single or no LRT.

**TABLE 4:** Multivariate logistic regression predictors of discordance.

Variables	Odds Ratio	95% CI		*p* value
Period				
Post‐October 2015 vs. Pre‐October 2015	**1.19**	**1.01**	**1.41**	**0.03**
Etiology				
ALD vs. HCV	0.92	0.66	1.27	0.13
Crypto vs. HCV	1.16	0.71	1.89	
HBV vs. HCV	1.62	0.95	2.77	
HCV_ALD vs. HCV	0.92	0.72	1.17	
NASH vs. HCV	1.28	0.92	1.78	
Age	1.01	1.00	1.02	0.28
Gender				
F vs. M	**0.71**	**0.60**	**0.85**	**0.0002**
Ethnicity				
Black vs. White	1.00	0.79	1.26	0.35
Hispanic vs. White	0.96	0.79	1.18	
Other vs. White	0.76	0.56	1.03	
Region				
Central vs. West	0.85	0.68	1.06	0.08
Northeast vs. West	**0.78**	**0.63**	**0.97**	
South vs. West	**0.71**	**0.57**	**0.87**	
Wait time in months	1.00	1.00	1.00	0.15
Allocation MELD	1.00	0.97	1.02	0.73
AFP				
21–99 vs. < =20	**1.48**	**1.25**	**1.77**	**<0.0001**
100–499 vs. < =20	**1.58**	**1.23**	**2.05**	
> = 500 vs. < =20	**2.89**	**1.75**	**4.75**	
BMI	1.00	1.00	1.02	0.64
Tumor location				
Left vs. both	**0.10**	**0.08**	**0.12**	**<0.0001**
Right vs. both	**0.20**	**0.18**	**0.24**	
Max tumor size (cm)				
1–2 vs. <1	0.87	0.71	1.05	<0.0001
2–3.5 vs. <1	**0.77**	**0.64**	**0.93**	
> = 3.5 vs. <1	**1.55**	**1.17**	**2.07**	
Number of tumors				
2 vs. 1	**1.34**	**1.07**	**1.69**	**0.0004**
3 vs. 1	**1.83**	**1.30**	**2.59**	
Treatments (LRT) since listing				
0 vs. >1	**0.54**	**0.46**	**0.65**	**<0.0001**
1 vs. >1	**0.54**	**0.42**	**0.71**	
Treatments (LRT) since last radiology				
0 vs. > −1	0.63	0.36	1.12	0.12
Thermal ablation				
No vs. yes	0.81	0.38	1.74	0.59
Chemoembolisation				
No vs. yes	1.45	0.79	2.65	0.23
HCV serostatus				
Positive vs. negative	1.05	0.80	1.37	0.47
Unknown/not disclosed vs. negative	0.69	0.35	1.36	
HBcAB				
Positive vs. negative	**1.17**	**1.00**	**1.37**	0.14
Unknown/not disclosed vs. negative	1.10	0.68	1.76	
HBsAg				
Positive vs. negative	0.94	0.59	1.49	0.15
Unknown/not disclosed vs. negative	0.57	0.32	1.01	
Imaging				
MRI vs. CT	1.14	0.99	1.32	0.07
Time from imaging to transplant (days)	**1.00**	**1.00**	**1.01**	**0.05**

Significant *p* values are indicated in bold.

Abbreviations: AFP, alpha‐fetoprotein; ALD, Alcohol related liver diseases; BMI, body mass index; HBcAB, hepatitis B core antibody; HBsAg, hepatitis B surface antigen; HBV, hepatitis B virus; HCV, hepatitis C virus; LRT, locoregional treatment; NASH, nonalcoholic steatohepatitis.

### 3.4 Post‐LT outcomes

HCC recurrence occurred in 6.26% patients after a median follow‐up of 364 (IQR: 193–626) days.

Group 1 had 4.1% recurrence while group 2 had 10.6% recurrence at 3 years post‐LT (*p* < 0.0001). Table S4.

**FIGURE 2:**
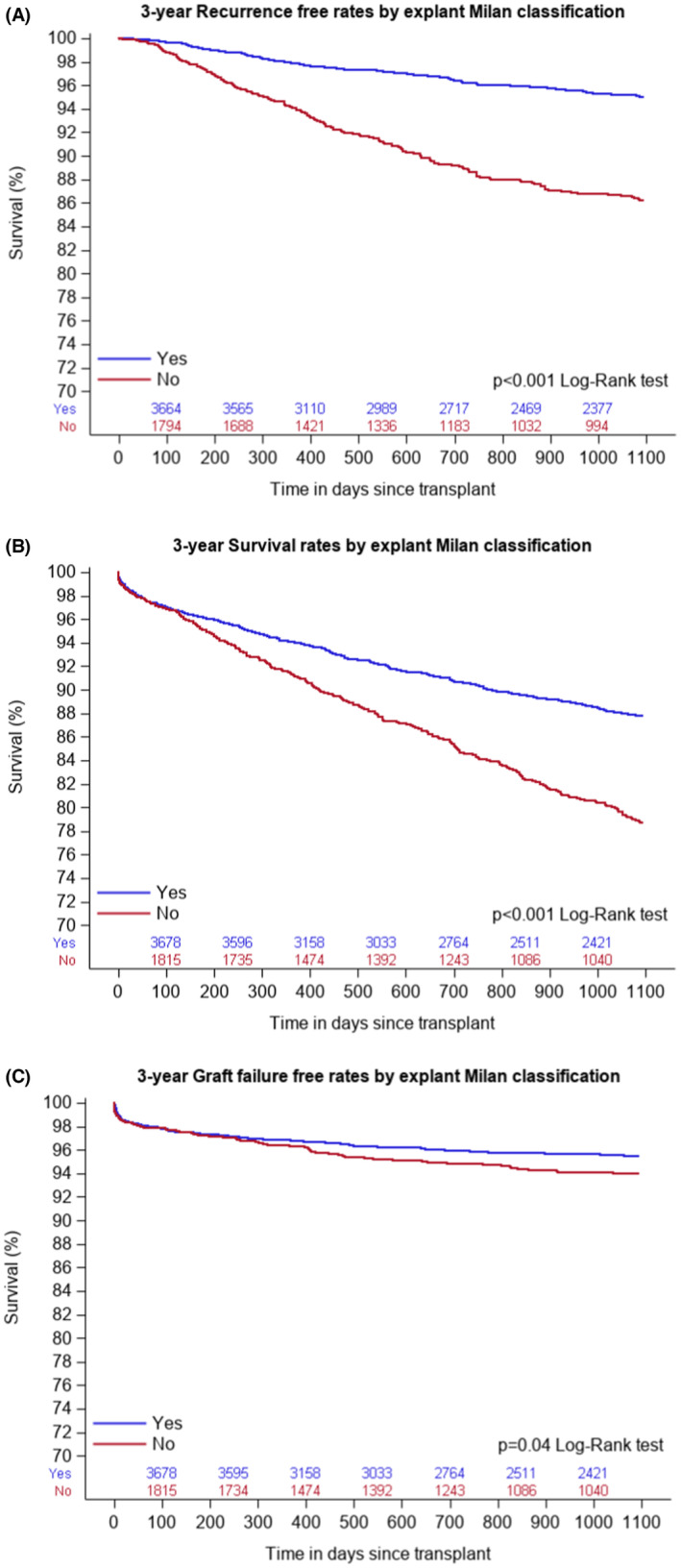
(A) Showing Kaplan–Meier curve for 3‐year recurrence by explant histopathology. (B) Showing Kaplan–Meier curve for 3‐year mortality by explant histopathology. (C) Showing Kaplan–Meier curve for 3‐year graft failure by explant histopathology.

As shown in Table 5, Being outside Milan on explant was associated with increased risk of recurrence (HR 1.86, 95% CI 1.32–2.63, *p* < 0.0001), and higher mortality (HR 1.32, 95% CI 1.03–1.70, *p* < 0.03) than inside Milan on explant. Figure 2A–C showed increased recurrence *p* < 0.001, decreased survival *p* < 0.001, and increased graft failure *p* = 0.04, respectively, in this group compared with inside Milan in explant.

**TABLE 5:** Multivariable Cox model for post‐LT outcomes: 3‐year recurrence, 3‐year mortality, and 3‐year graft failure in correlation with patients' characteristics and era.

Variables		3‐year recurrence		3‐year mortality		3‐year graft failure	
		HR (95% CI)	*p* value	HR (95% CI)	*p* value	HR (95% CI)	*p* value
Explant within Milan	No vs. yes	**1.86 (1.32, 2.63)**	**<0.001**	**1.32 (1.03, 1.70)**	**0.03**	0.97 (0.62, 1.50)	0.88
6 months wait time policy	Post vs. pre	0.88 (0.63, 1.22)	0.44	1.11 (0.89, 1.38)	0.36	0.93 (0.66, 1.33)	0.71
Etiology	HBV/HCV	Ref		Ref		Ref	
	ALD	1.01 (0.53, 1.93)	0.98	**0.54 (0.36, 0.83)**	**0.01**	0.54 (0.25, 1.16)	0.12
	Crypto	1.24 (0.53, 2.89)	0.62	**0.42 (0.23, 0.80)**	**0.01**	0.69 (0.24, 2.01)	0.50
	HBV	0.81 (0.29, 2.30)	0.69	**0.30 (0.14, 0.62)**	**0.001**	0.47 (0.15, 1.52)	0.21
	HCV	1.22 (0.79, 1.87)	0.38	**0.72 (0.56, 0.94)**	**0.02**	0.68 (0.45, 1.03)	0.07
	NASH	0.54 (0.26, 1.14)	0.11	**0.53 (0.35, 0.82)**	**0.00**	0.61 (0.28, 1.31)	0.21
Age		1.00 (0.98, 1.02)	0.93	**1.02 (1.01, 1.04)**	**0.00**	0.99 (0.97, 1.01)	0.29
Gender	Female vs. Male	**0.68 (0.49, 0.95)**	**0.02**	0.88 (0.72, 1.09)	0.25	0.98 (0.69, 1.39)	0.93
Ethnicity	White	Ref		Ref		Ref	
	Black	0.92 (0.64, 1.32)	0.64	1.16 (0.90, 1.48)	0.25	**1.56 (1.06, 2.28)**	**0.02**
	Hispanic	0.94 (0.68, 1.30)	0.69	0.89 (0.70, 1.14)	0.36	**0.60 (0.37,0.99)**	**0.05**
	Other	0.85 (0.49, 1.46)	0.56	1.02 (0.70, 1.47)	0.92	1.03 (0.57, 1.87)	0.92
Region	West	Ref		Ref		Ref	
	Central	0.93 (0.63, 1.36)	0.69	1.10 (0.85, 1.43)	0.46	1.09 (0.70, 1.70)	0.70
	Northeast	0.92 (0.64, 1.33)	0.67	1.10 (0.85, 1.43)	0.47	1.10 (0.71, 1.71)	0.67
	South	1.15 (0.81, 1.64)	0.44	1.06 (0.83, 1.36)	0.63	0.79 (0.51, 1.23)	0.30
Functional status	High	Ref		Ref		Ref	
	Low	1.23 (0.87, 1.74)	0.25	1.26 (0.97, 1.63)	0.08	1.22 (0.77, 1.93)	0.40
	Moderate	1.00 (0.78, 1.29)	0.98	1.20 (1.00, 1.43)	0.05	1.28 (0.94, 1.75)	0.12
	Unknown	1.30 (0.31, 5.47)	0.72	0.73 (0.18, 2.95)	0.66	1.04 (0.14, 7.61)	0.97
Wait time (months)		0.99 (0.98, 1.00)	0.17	1.00 (0.99, 1.01)	0.22	1.00 (0.99, 1.01)	0.77
Donor risk index		0.99 (0.70, 1.41)	0.96	**1.37 (1.08, 1.74)**	**0.01**	**2.22 (1.51, 3.25)**	**<0.0001**
MELD at allocation		**1.05 (1.01, 1.09)**	**0.01**	0.99 (0.98, 1.00)	0.19	**0.97 (0.96, 0.98)**	**<0.0001**
AFP	<=20	Ref		Ref		Ref	
	21–99	**1.65 (1.24, 2.18)**	**0.001**	**1.49 (1.23, 1.80)**	**<0.0001**	1.20 (0.85, 1.69)	0.30
	100–499	**2.63 (1.91, 3.62)**	**<0.0001**	**1.48 (1.14, 1.94)**	**0.004**	1.12 (0.69, 1.82)	0.64
	> = 500	**3.22 (1.99, 5.22)**	**<0.0001**	**2.60 (1.74, 3.90)**	**<0.0001**	1.55 (0.68, 3.57)	0.30
DCD Donor		0.69 (0.45, 1.06)	0.09	**0.72 (0.54, 0.95)**	**0.02**	0.72 (0.45, 1.13)	0.15
Donor HT	No	Ref		Ref		Ref	
	Unknown	0.83 (0.12, 6.01)	0.86	1.68 (0.74, 3.79)	0.21	**2.97 (1.07, 8.23)**	**0.04**
	Yes	1.16 (0.90, 1.49)	0.25	**1.14 (0.95, 1.36)**	0.15	**1.45 (1.07, 1.96)**	**0.02**
Worst tumor differentiation	Well	Ref		Ref		Ref	
	Complete tumor necrosis	0.71 (0.41, 1.22)	0.21	0.84 (0.63, 1.13)	0.26	0.61 (0.37, 1.01)	0.07
	Moderate	**2.25 (1.57, 3.24)**	**<0.0001**	**1.34 (1.08, 1.65)**	0.01	1.28 (0.91, 1.79)	0.15
	Poor	**6.50 (4.33, 9.75)**	**<0.0001**	**2.84 (2.17, 3.72)**	<0.0001	**2.06 (1.26, 3.38)**	**0.00**
Max tumor size		**1.12 (1.06, 1.18)**	**<0.0001**	**1.07 (1.01, 1.12)**	0.01	1.05 (0.96, 1.15)	0.29
Number of tumors	>5	Ref		Ref		Ref	
	1	**0.55 (0.35, 0.86)**	**0.01**	**0.57 (0.40, 0.82)**	**0.00**	0.92 (0.44, 1.93)	0.83
	2	**0.48 (0.32, 0.73)**	**0.001**	**0.56 (0.40, 0.78)**	**0.00**	1.00 (0.50, 2.02)	0.99
	3	**0.35 (0.21, 0.57)**	**<0.0001**	**0.54 (0.38, 0.78)**	**0.00**	0.96 (0.45, 2.01)	0.90
	4	**0.41 (0.25, 0.67)**	**0.000**	**0.51 (0.35, 0.75)**	**0.00**	0.74 (0.32, 1.73)	0.49
	5	**0.57 (0.34, 0.98)**	**0.04**	0.65 (0.42, 1.00)	**0.05**	1.24 (0.52, 2.94)	0.62
Treatments since listing	1+	Ref		Ref		Ref	
	0	**1.43 (1.03, 2.00)**	**0.03**	0.97 (0.77, 1.21)	0.76	0.71 (0.49, 1.01)	0.06
	1	0.94 (0.57, 1.54)	0.81	1.04 (0.77, 1.39)	0.81	0.64 (0.39, 1.04)	0.07
Thermal ablation	No vs. yes	0.72 (0.31, 1.67)	0.44	1.42 (0.63, 3.21)	0.40	2.96 (0.41, 21.33)	0.28
Chemoembolization	No vs. yes	1.09 (0.73, 1.63)	0.67	0.91 (0.69, 1.19)	0.49	1.41 (0.82, 2.42)	0.21
TIPSS	No	Ref		Ref		Ref	
	Unknown	1.08 (0.32, 3.57)	0.91	0.66 (0.21, 2.06)	0.47	0.77 (0.11, 5.55)	0.80
	Yes	**0.44 (0.21, 0.92)**	**0.03**	0.94 (0.67, 1.33)	0.73	0.71 (0.36, 1.41)	0.33
HCV serostatus	Negative	Ref		Ref		Ref	
	Positive	0.75 (0.49, 1.15)	0.19	**0.68 (0.51, 0.90)**	**0.01**	0.93 (0.54, 1.59)	0.79
	Unknown/not disclosed	**0.22 (0.05, 0.90)**	0.04	0.40 (0.15, 1.10)	0.08	2.08 (0.67, 6.48)	0.21
HBcAB	Negative	Ref		Ref		Ref	
	Positive	1.22 (0.94, 1.58)	0.13	1.04 (0.87, 1.25)	0.68	1.13 (0.83, 1.54)	0.43
	Unknown/not disclosed	1.08 (0.47, 2.50)	0.85	1.00 (0.54, 1.85)	0.99	0.81 (0.26, 2.51)	0.72
HBsAg	Negative	Ref		Ref		Ref	
	Positive	1.38 (0.62, 3.10)	0.43	1.57 (0.91, 2.73)	0.11	1.10 (0.44, 2.75)	0.84
	Unknown/not disclosed	1.32 (0.59, 2.96)	0.50	0.55 (0.24, 1.23)	0.14	0.55 (0.15, 1.98)	0.36
Graft failure before outcome of interest	Yes vs. no	**5.67 (1.92, 16.79)**	**0.00**	**11.62 (6.26, 21.55)**	**<0.0001**		

Significant *p* values are indicated in bold.

Abbreviations: AFP, alpha‐fetoprotein; ALD, Alcohol related liver diseases; BMI, body mass index; DCD donor, donor after cardiac death; HBcAB, hepatitis B core antibody; HBsAg, hepatitis B surface antigen; HBV, Hepatitis B virus; HCV, hepatitis C virus; HT, hypertension; LRT, locoregional treatment; MELD, model of end‐stage liver disease; NASH, nonalcoholic steatohepatitis.

Males had increased risk of recurrence (HR 0.68, 95% CI 0.49–0.95, *p* < 0.023). Advancing age was associated with increased mortality (HR 1.02, 95% CI 1.01–1.04, *p* < 0.001). AFP >500 ng/mL was associated with high recurrence and increased mortality (HR 3.22, 95% CI 1.99–5.22, *p* < 0.0001) and (HR 2.60, 95% CI 1.74–3.90, *p* < 0.0001), respectively. Poor tumor differentiation was associated with increased recurrence and mortality (HR 6.50, 95% CI 4.33–9.75, *p* < 0.001) and (HR 2.84, 95% CI 2.17–3.72, *p* < 0.001), respectively. A higher tumor diameter was associated with increased recurrence and mortality (HR 1.12, 95% CI 1.06–1.18, *p* < 0.0001) and (HR 1.07, 95% CI 1.01–1.12, *p* = 0.012), respectively. Receiving no LRT pre‐LT led to increased risk of recurrence (HR 1.43, 95% CI 1.03–2.00, *P* = 0.03). Predictors of 3‐year graft failure include higher donor risk index (DRI), *p* < 0.0001, donors with hypertension, *P* = 0.02, and poor tumor differentiation, *p* = 0.004.

There was no change in post‐LT outcomes after adjusting for the period of the 6‐month waiting policy (before or after).

### 3.5 Contrast‐enhanced liver protocol imaging techniques (CT vs. MRI) and discordance

There was no statistically significant difference between the diagnostic yield of contrast‐enhanced CT or contrast‐enhanced MRI in agreement with explant pathology, *p* = 0.18. There was no statistically significant difference between CT and MRI in the detection of tumor size (*p* < 0.07), total tumor diameter (*p* = 0.07), and the number of tumors in imaging versus explant (*p* = 0.37). Table 6 For patients within Milan on their most recent pre‐LT radiology, the PPV for concordance of MRI was 67.13%, while that of CT was 68.67%. Table S5 and S6 A multivariable analysis to detect the effect of imaging technique on the 3‐year recurrence showed no statistical difference between CT and MRI in predicting recurrence (HR 1.14; 95% CI 0.99–1.32). Table 4.

**TABLE 6:** The diagnostic yield of HCC between pre‐LT Radiology techniques and explant histopathology.

	CT	MRI	*p* value
Difference in tumor size if 1 tumor only, mean (SD) between radiology and explant histopathology	1.44 (1.92)	1.55 (1.92)	0.07
Difference in max size if < =3 tumors, mean (SD) between radiology and explant histopathology	1.53 (1.94)	1.64 (2.19)	0.07
Difference in number of tumors, *N* (%) between radiology and explant histopathology			0.37
0 (no diff.)	1118 (52.4)	2076 (52.6)	
1	526 (24.65)	965 (24.45)	
2	262 (12.28)	427 (10.82)	
3	121 (5.67)	252 (6.38)	
4	107 (5.01)	227 (5.75)	

## 4 DISCUSSION

In the largest to date study cohort of 6842 patients with HCC with LT, we found discordant results between pre‐LT radiology (within 90 days) and histopathology in 33.3% of study subjects. The Median time from last imaging to liver transplantation was 35 days (15–59). Male gender, higher number (>1) of tumor, larger size (>3.5 cm), bilaterality of tumors, increasing AFP (>20 ng/mL), and treatment with LRT were predictors of radiological‐histopathological discordance. The discordance led to significantly increased 3‐year mortality and recurrence. In addition, graft allocation policy with 6‐month waiting time implemented in 2015 led to increased discordance, although it did not impact post‐LT outcomes. We found no difference between contrast‐enhanced liver protocol CT and MRI in predicting the discordance.

In our study, we collected data for tumor number, size and presence or absence of vascular invasion in the explant histopathology to correlate Milan stage in pre‐LT imaging. Previous reports attributed discordance between pre‐LT scans and explant histopathology to multiple factors, including nonstandardized sectioning techniques, differences in sensitivity and specificity of imaging modalities, and the affinity of tumors to contrast material.^9^, ^10^, ^11^, ^12^, ^13^ The Liver Imaging and Reporting Data System (LIRADS) was introduced 2018 to increase accuracy of assessing indeterminate lesions and to improve consistency among radiologists. However, discordance in LIRADS observations was found comparing MRI reporting between radiologists in 42% for LIRADS 5 lesions and 60% in LIRADS 3 and 4 lesions.^14^ Cunha et al, attributed discordance to dissimilarity between T2 lesions in radiology in correlation to T2 in histopathology. Linking explant histopathology to pre‐LT radiology is challenging as histopathological T2 staging includes vascular invasion (microvascular or small vessel) or multiple lesions up to 5 cm.^15^ Another report by Ecker, et al. described multifocality of the tumor and multiplicity of lesions as predictors for discordance between MRI pre‐LT and explant.^16^ A recently published report identified center level variation between different UNOS regions as a risk factor for discordance. The rate of discordance ranged from <20% in some regions to >30% in other regions. The authors attributed the discordance to behavioral bias in reporting between different regions.^9^

Liver transplantation is a curative option for HCC management with a good outcome and plausible survival benefit (75% at 5 years).^5^ Post‐LT recurrence and mortality are affected by tumor biology, behavior, and response to pre‐LT LRT.^17^ The recurrence of HCC after LT remains a problem with mean recurrence rate of 16% in just over 1 year.^18^ In our study, recurrence rate was 4% in patients within Milan by explant histopathology and 10% if explant histopathology showed features outside Milan. Emphasizing the importance of tumor biology and behavior we found that poor tumor differentiation, and multiplicity of tumors were associated with increased 3‐year mortality and recurrence. This emphasizes the importance of explant pathology in prediction of post‐LT mortality and recurrence. Increasing AFP and AFP > 20 ng/mL were also associated with increased recurrence and mortality. Earlier report by Mehta et al. described AFP > 100 ng/mL at LT as a predictor for recurrence and mortality. The authors emphasized AFP for patients undergoing LRT down‐staging as an independent risk factor for recurrence.^19^ Moreover, our analysis showed increased recurrence in patients who had no LRT on wait‐list. Kim et al. reported pathologic down‐staging by LRT as a predictor of higher survival.^20^

Since 2002, the United States adopted MELD‐based allocation system to liver transplantation to decrease wait‐list mortality. However, HCC patients were subjected to increased mortality on the waiting list as MELD score alone did not allow for a timely transplantation.^7^ UNOS adopted the MELD exception points for patients with standard T1 and T2 lesions to mitigate a 15% increased risk of 3‐month dropout rate. The exception points should be increased every 3 months to account for 10% increased risk of mortality if transplantation did not take place.^21^ The latter led to overprioritization of HCC patients as compared with their matching MELD non‐HCC transplant candidate especially in centers with higher volume.^7^ A previous analysis of the UNOS database showed that under original MELD exception policy, HCC patients had higher odds to get transplanted (OR = 1.6, *p* < 0.001) and lower odds of wait‐list dropout (OR = 0.47, *p* < 0.001) as compared with non‐HCC patients.^22^

To optimize organ allocation and improve post‐LT outcome, UNOS/ OPTN (Organ Procurement and Transplantation Network) implemented the 6‐month waiting policy. The mandatory wait time allowed observation of tumor behavior, and locoregional treatment to reduce tumor burden.^23^, ^24^ The policy was in balancing the risk of wait‐list dropout/mortality between HCC and non‐HCC patients; however, the substantial advantage of HCC policy remained the same.^25^ Moreover, the policy succeeded to homogenize the median time from registration to first transplantation between different UNOS regions. However, after implementation of the policy there was a notable increase in number of tumors and wait‐list LRTs as shown by previous analysis by Durkin et al.^26^ Our data showed that after implementation of the policy there was a trend of transplanting patients with a higher AFP and an increased odds of being outside Milan on explant, yet there was a decrease in overall tumor burden due to necrosis. Furthermore, the volume of patients transplanted with HCC decreased among all regions between the two eras, which support the goal of the policy to overcome the imbalance of access to transplant between HCC and non‐HCC patients.^23^ However, there was no statistically significant difference in the 3‐year recurrence and mortality between the two eras. This finding is similar to previous data describing no effect of the new policy on post‐LT outcomes.^8^

Earlier reports emphasized the reliability of MRI in detection of small or indeterminate lesions compared to CT.^27^ UNOS/OPTN led efforts to standardize diagnostic criteria for HCC imaging between different diagnostic modalities.^15^ Our study showed no statistical difference between contrast‐enhanced CT and MRI in predicting radiological‐histopathological discordance. Newer imaging techniques incorporating artificial intelligence may overcome the discordance. Noncontrast‐enhanced MRI radiomics signature is a promising imaging method with potential utility in detection of histological grades, MVI and response to local and systemic therapies.^28^

Our study had limitations using a large heterogeneous national database with a potential risk of nondifferential misclassification bias. Moreover, some patients did not have pretransplant imaging in their records, and some had their post‐LRT assessment by ultrasound only. To address that, we excluded records with missing CT/MRI pre‐LT from our analysis. The LIRADS system was adopted to alleviate discordance in reporting of HCC.^14^ However, in UNOS/OPTN database there is no standardized reporting system by histopathologists. Furthermore, HCC recurrence has multiple risk factors, and some of the risk factors cannot be fully explored due to incomplete reports and differences in reporting techniques between different regions. Nonetheless, our study with a large sample size showed a greater magnitude of discordance than previously reported.

In conclusion, the results of our study showed that staging of HCC based on current radiological techniques has a high discordance rate and leads to underestimation of HCC burden in one out of three patients. It accounts significantly for increased post‐LT HCC recurrence and mortality. The implementation of 6‐month waiting time policy led to increased discordance. Patients with risk factors for discordance may need enhanced surveillance and aggressive LRT to achieve complete tumor necrosis to improve post‐LT outcomes. Improvement in radiological assessment with possible incorporation of artificial intelligence is needed to identify patients at risk for discordance.

## AUTHOR CONTRIBUTIONS

**Mohamed Ismail:** Conceptualization (lead); formal analysis (equal); investigation (equal); methodology (equal); writing – original draft (supporting); writing – review and editing (supporting). **Ahmed El Sabagh:** Writing – original draft (equal); writing – review and editing (equal). **Ahmed M. Afifi Abdelwahab:** Writing – review and editing (equal). **Efstathia Polychronopoulou:** Formal analysis (lead); methodology (lead); writing – original draft (supporting); writing – review and editing (supporting). **Yong‐Fang Kuo:** Investigation (lead); methodology (lead); supervision (equal); writing – original draft (equal); writing – review and editing (equal). **Manal Hassan:** Methodology (equal); supervision (equal); writing – original draft (equal); writing – review and editing (equal). **John Goss:** Supervision (lead); writing – original draft (equal); writing – review and editing (lead). **Fasiha Kanwal:** Supervision (lead); writing – original draft (equal); writing – review and editing (lead). **Prasun K Jalal:** Conceptualization (lead); formal analysis (equal); investigation (equal); methodology (equal); supervision (lead); writing – original draft (lead); writing – review and editing (lead).

## CONFLICT OF INTEREST STATEMENT

There is none to declare for all authors related to the current study.

## ETHICS STATEMENT

Approval of the research protocol by an Institutional Reviewer Board: This study was conducted under IRB approval of Baylor College of medicine. Informed Consent: Consent waiver to unidentified data from UNOS registry.

## Supporting information


Tables S1‐S6

## Data Availability

Guidelines for General Public: In order for the general public to submit a request, the requester will need to go to: https://optn.transplant.hrsa.gov/data/request‐data/ and fill out a formal request for this information. Guidelines for OPTN Members: OPTN Members may request data they have previously submitted at any time, without charge. OPTN Members with UNetSM access may submit a request by logging into UNetSM, scrolling to the bottom of the page and clicking “add a New Request.” OPTN Members without UNetSM access can submit requests through the same link on the OPTN website as described above for the general public: https://optn.transplant.hrsa.gov/data/request‐data/
