# Supplementary material for: Radiological‐histopathological discordance in patients transplanted for HCC and its impact on post‐transplant outcomes
Source: Cancer Med. 2023 Jun 16;12(14):15011–25. doi: 10.1002/cam4.6161 (PMC10417193; doi:10.1002/cam4.6161)
Supplement: Supplementary file 1 — Tables S1‐S6 [file CAM4-12-15011-s001.docx]

***Supplemental tables:***

**Table 1:** Transplant-related clinical data in transplanted patients with HCC within Milan by pre-LT radiology (Group 1 and 2), not included in Table 2:

|  | | **N** | **Mean** | **St. Dev** | **Median** | **Q1** | **Q3** | **p-value** |
| --- | --- | --- | --- | --- | --- | --- | --- | --- |
| **Variables** | **Milan at explant** | 2278 | 43.9 | 15.7 | 44.0 | 30.0 | 56.0 | 0.63 |
| **Donor Age** | No |  |  |  |  |  |  |  |
|  | Yes | 4564 | 44.1 | 15.4 | 44.0 | 31.0 | 56.0 |  |
| **Donor BMI** | No | 2276 | 28.4 | 6.5 | 27.2 | 23.8 | 31.6 | 0.97 |
|  | Yes | 4555 | 28.3 | 6.5 | 27.2 | 23.9 | 31.6 |  |
| **Macro fat (%)** | No | 935 | 8.8 | 11.5 | 5.0 | 0.0 | 10.0 | 0.42 |
|  | Yes | 1997 | 8.5 | 12.3 | 5.0 | 0.0 | 10.0 |  |
| **Cold ischemic time** | No | 2268 | 6.3 | 2.5 | 6.0 | 4.8 | 7.6 | **0.01** |
|  | Yes | 4556 | 6.2 | 2.4 | 5.9 | 4.6 | 7.4 |  |
| **Donor Risk Index** | No | 2268 | 1.5 | 0.4 | 1.4 | 1.1 | 1.7 | 0.84 |
|  | Yes | 4556 | 1.4 | 0.4 | 1.4 | 1.1 | 1.7 |  |
| **Recipient age** | No | 2278 | 61.0 | 6.2 | 61.0 | 57.0 | 65.0 | 0.18 |
|  | Yes | 4564 | 60.7 | 6.1 | 61.0 | 57.0 | 65.0 |  |
| **Recipient BMI** | No | 2278 | 29.2 | 5.1 | 28.6 | 25.6 | 32.2 | 0.38 |
|  | Yes | 4564 | 29.1 | 5.0 | 28.6 | 25.4 | 32.3 |  |
| **Allocation meld score** | No | 2270 | 27.7 | 6.1 | 28.0 | 25.0 | 31.0 | **0.01** |
|  | Yes | 4556 | 27.6 | 3.9 | 28.0 | 25.0 | 31.0 |  |
| **Meld lab score** | No | 2278 | 12.2 | 5.2 | 11.0 | 8.0 | 15.0 | 0.07 |
|  | Yes | 4564 | 11.8 | 4.7 | 11.0 | 8.0 | 14.0 |  |
| **Wait time (months)** | No | 2278 | 12.6 | 16.2 | 8.9 | 4.9 | 14.9 | 0.36 |
|  | Yes | 4564 | 13.2 | 18.3 | 8.4 | 4.5 | 15.1 |  |
| **AFP, continuous** | No | 1918 | 62.9 | 346.9 | 9.0 | 5.0 | 28.0 | **<.0001** |
|  | Yes | 4020 | 38.9 | 213.5 | 7.0 | 4.0 | 17.0 |  |
| **Largest tumor size from radiology** | No | 1048 | 2.3 | 0.9 | 2.1 | 1.6 | 2.8 | 0.52 |
|  | Yes | 2192 | 2.2 | 0.8 | 2.1 | 1.6 | 2.7 |  |
| **Largest tumor size from explant** | No | 2238 | 4.1 | 2.0 | 3.8 | 3.0 | 5.1 | **<.0001** |
|  | Yes | 4564 | 2.3 | 1.0 | 2.2 | 1.6 | 3.0 |  |
| **Total tumor size from radiology** | No | 1048 | 2.9 | 1.3 | 2.7 | 1.9 | 3.8 | **<.0001** |
|  | Yes | 2192 | 2.6 | 1.1 | 2.4 | 1.8 | 3.2 |  |
| **Total tumor size from explant** | No | 2238 | 7.2 | 3.0 | 6.5 | 5.3 | 8.4 | **<.0001** |
|  | Yes | 4564 | 2.8 | 1.3 | 2.7 | 1.9 | 3.7 |  |

**Table 2:** Explant Histopathology before and after 6-month waiting policy.

|  | **N** | **Explant**  **Within Milan (%)** | **Explant**  **Outside Milan (%)** | **p-value** |
| --- | --- | --- | --- | --- |
| **Pre-policy** | 4328 | 2950 (68.2) | 1378 (31.8) | **<0.0015** |
| **Post-policy** | 1455 | 926 (63.6) | 529 (36.4) |  |

**Table 3:** The effect of 6-month waiting policy on discordance by region.

| Region | 6-month waiting policy | **N** | **Explant**  **Within Milan (%)** | **Explant**  **Outside Milan (%)** | **p-value** |
| --- | --- | --- | --- | --- | --- |
| Central | Pre-policy | 884 | 599 (67.8) | 285 (32.2) | 0.19 |
|  | Post-policy | 340 | 217 (63.8) | 123 (36.2) |  |
| Northeast | Pre-policy | 1112 | 759 (68.3) | 353 (31.7) | 0.001 |
|  | Post-policy | 348 | 206 (59.2) | 142 (40.8) |  |
| South | Pre-policy | 1420 | 1017 (71.6) | 403 (28.4) | 0.31 |
|  | Post-policy | 494 | 342 (69.2) | 152 (30.8) |  |
| West | Pre-policy | 912 | 575 (63.1) | 337 (36.9) | 0.22 |
|  | Post-policy | 273 | 161 (59.0) | 112 (41.0) |  |

**Table 4:** Tumor recurrence rates at 3-yrs in different groups of Study Population.

|  |  | **Recurrence** | | |  |
| --- | --- | --- | --- | --- | --- |
| **Radiology** | **Explant** | Yes | No | Total | p-value |
| Milan: Yes | Milan: Yes (Group 1) | 186 (4.08) | 4378 (95.92) | 4564 (66.71) | **<.0001** |
|  | Milan: No  (Group 2) | 242 (10.62) | 2036 (89.38) | 2278 (33.29) |  |
| Total |  | 428 (6.26) | 6414 (93.74) | 6842 |  |
| Milan: No | Milan: Yes  (Group 3) | 2 (3.23) | 60 (96.77) | 62 (40.79) | **0.02** |
|  | Milan: No  (Group 4) | 13 (14.44) | 77 (85.56) | 90 (59.21) |  |
| Total |  | 15 (9.87) | 137 (90.13) | 152 |  |

**Table 5:** The percentage of agreement between MRI and Histopathology.

|  | **Explant** | | |  |
| --- | --- | --- | --- | --- |
| **Radiology** | Milan: Yes (%) | Milan: No (%) | Total | p-value |
| Milan: Yes | 2587 (67.13) * | 1267 (32.87) | 3854 | **<.0001** |
| Milan: No | 41 (44.09) | 52 (55.91) | 93 |  |
| Total | 2628 | 1319 | 3947 |  |

*PPV = 67.13%

**Table 6:** The percentage of agreement between CT and Histopathology:

|  | **Explant** | | |  |
| --- | --- | --- | --- | --- |
| **Radiology** | Milan: Yes (%) | Milan: No (%) | Total | P value |
| Milan: Yes | 1425 (68.67) * | 650 (31.33) | 2075 | **<.0001** |
| Milan: No | 21 (35.59) | 38 (64.41) | 59 |  |
| Total | 1446 | 688 | 2134 |  |

*PPV = 68.67%
